# The media and intellectuals' response to medical publications: the antidepressants' case

**DOI:** 10.1186/1744-859X-12-11

**Published:** 2013-04-12

**Authors:** Konstantinos N Fountoulakis, Cyril Hoschl, Siegfried Kasper, Juan Lopez-Ibor, Hans-Jürgen Möller

**Affiliations:** 1 3rd Department of Psychiatry, School of Medicine, Aristotle University of Thessaloniki, 54636, Thessaloniki, Greece; 2 Department of Psychiatry and Medical Psychology, Prague Psychiatric Center, 18103, Prague, Czech; 3 Charles University, 18103, Prague, Czech Republic; 4 Department of Psychiatry and Psychotherapy, Medical University of Vienna, 1090, Vienna, Austria; 5 Institute of Psychiatry and Mental Health, 28035, Madrid, Spain; 6 WHO Collaborating Centre for Research and Training in Mental Health and Health Research Institute, Instituto de Investigación Sanitaria San Carlos (IdISSC), 28035, Madrid, Spain; 7 Center for Biomedical Research Network on Mental Health (CIBERSAM), Hospital Clínico San Carlos, 28035, Madrid, Spain; 8 Department of Psychiatry, Faculty of Medicine, Universidad Complutense, 28035, Madrid, Spain; 9 Department of Psychiatry, Ludwig Maximilians University, 80336, Munich, Germany

**Keywords:** Antidepressants, Efficacy, Debate, Media, Lay persons

## Abstract

During the last decade, there was a debate concerning the true efficacy of antidepressants. Several papers were published in scientific journals, but many articles were also published in the lay press and the internet both by medical scientists and academics from other disciplines or representatives of societies or initiatives. The current paper analyzes the articles authored by three representative opinion makers: one academic in medicine, one academic in philosophical studies, and a representative of an activists' group against the use of antidepressants. All three articles share similar gaps in knowledge and understanding of the scientific data and also are driven by an ‘existential-like’ ideology. In our opinion, these articles have misinterpreted the scientific data, and they as such may misinform or mislead the general public and policy makers, which could have a potential impact upon public health. It seems that this line of thought represents another aspect of the stigma attached to people suffering from mental illness.

## Introduction

**Figure 1:**
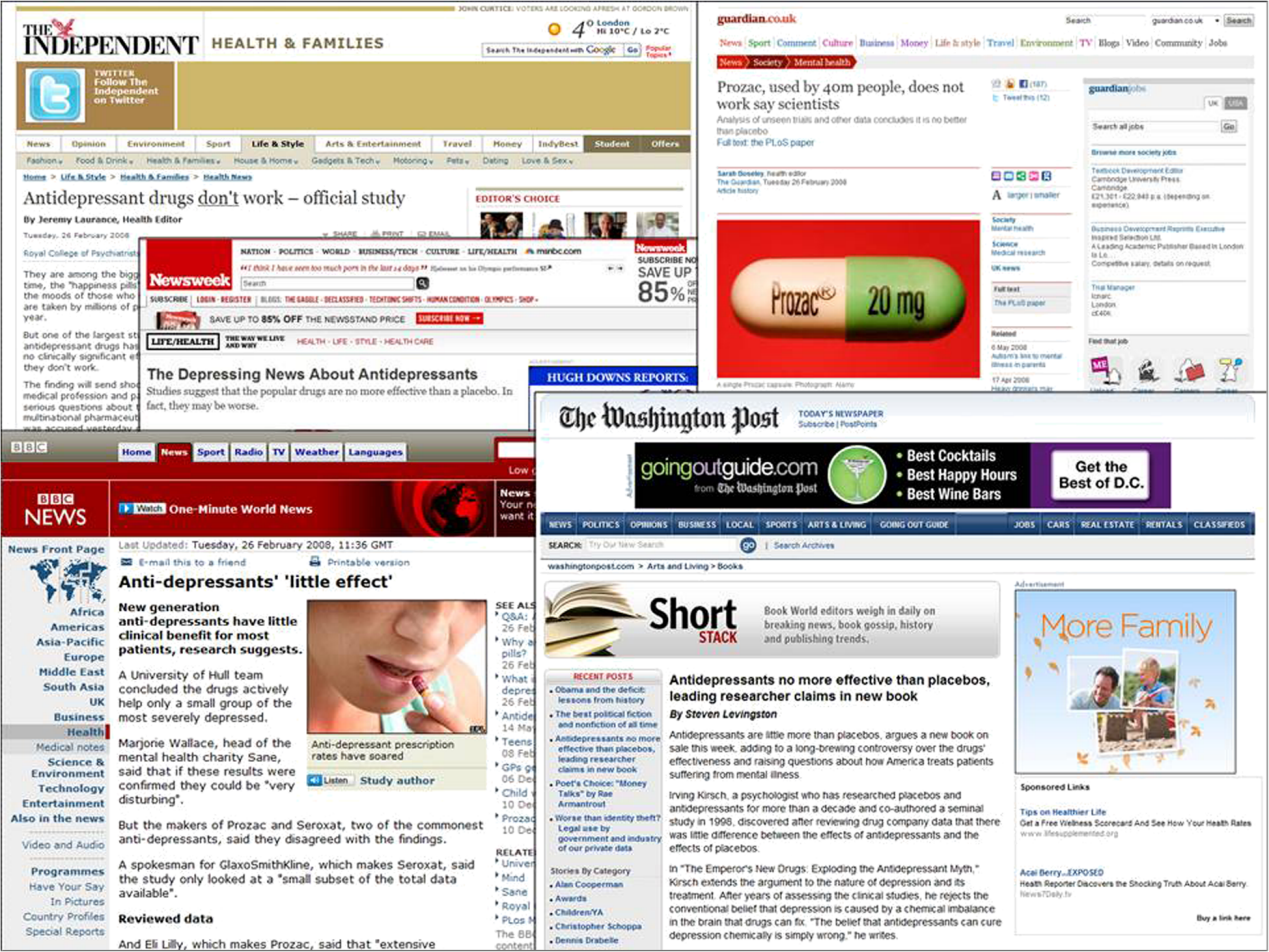
The depiction of the debate over the efficacy of antidepressants in high-reputation newspapers.

Recently, a number of meta-analytic studies disputed the clinical usefulness of antidepressants by reporting that their effect size is small [1-6] and that there is a significant bias in the publication of antidepressant trials [7]. These conclusions attracted much attention both by scientists and by the general public (see list of sites below, Figure 1). At the center of this debate, there was the meta-analysis by Kirsch et al. [4] which suggested that antidepressants fall well below criteria for clinical relevance and that efficacy reaches clinical relevance only in trials involving the most severely depressed patients. Kirsch went further and accused the Food and Drug Administration (FDA) as having an explicit decision to keep this information from the public and from prescribing physicians [8]. The Kirsch et al. data set [4] has been re-analyzed by two other groups [9,10], which independently reported results different to those reported by Kirsch et al. The interpretations also differed.

The list of sites of mass media which covered the antidepressants' debate soon after the Kirsch et al. 2008 publication:

•http://www.huffingtonpost.com/irving-kirsch-phd/antidepressants-the-emper_b_442205.html

•http://www.dana.org/news/cerebrum/detail.aspx?id=28024

•http://www.drugawareness.org/recentcasesblog/dr-irving-kirsch-exploding-the-antidepressant-myth

•http://www.newyorker.com/arts/critics/atlarge/2010/03/01/100301crat_atlarge_menand

•http://depression.about.com/library/weekly/aa072202.htm.

•http://www.independent.co.uk/life-style/health-and-families/health-news/antidepressant-drugs-udontu-work-ndash-official-study-787264.html

•http://entertainment.timesonline.co.uk/tol/arts_and_entertainment/the_tls/article7165698.ece.

•http://www.psychologytoday.com/blog/charting-the-depths/201002/listening-prozac-hearing-placebo

•http://www.guardian.co.uk/society/2008/feb/26/mentalhealth.medicalresearch

•http://discovermagazine.com/2008/oct/10-are-antidepressant-drugs-actually-worth-taking

•http://www.forbes.com/2010/01/05/antidepressant-paxil-placebo-business-healthcare-depression.html

•http://www.newsweek.com/2010/01/28/the-depressing-news-about-antidepressants.html

•http://www.aarp.org/health/drugs-supplements/info-05-2010/do_antidepressantswork.html

Furthermore, by ‘overstretching’ the interpretation of the data, Kirsch also suggested that because they do not incur drug risks, alternative therapies (e.g., exercise and psychotherapy) may be a better treatment choice for depression [8] and went on to author a book under the title *The Emperor's New Drugs: Exploding the Antidepressant Myth*[11].

In this frame, it is indeed peculiar that nobody comments on the finding of another meta-analysis which suggests that selective serotonin reuptake inhibitors (SSRIs) differ from psychotherapy as much as they differ from placebo (0.28 vs. 0.32) [12].

### Aim and methods

In this article, we will try to shed light on this issue. An important problem is that there are many technical details the average reader who is not a researcher or deeply involved in the field of mental health will find difficult to understand and follow. We will try to keep the arguments in a frame accessible to the average reader, and where necessary, we will provide additional information.

There are a number of articles in relationship to the Kirsch et al. paper on antidepressants in the lay press, written by persons established in their field. Three of them were selected for discussion in the current paper, as being quite representative. The choice was done according to the judgment of the authors of the current paper, and no strict criteria were applied. One (double article) is from a prominent academic in medicine, the second is from an academic outside medicine, and the third reflects the anti-medication and anti-psychiatric cultural movement. It is not possible to tackle all the points raised in these articles; however, some are overwhelmingly problematic, and they will be mentioned below. Of course, it is important to note also that some of the arguments are essentially right although ‘overstretched,’ e.g., the issue of conflict of interest or the problems in the diagnosis and classification of mental illness and its separation from normal conditions (e.g., grief). Also, an effort will be made to clarify the similarities and differences among them and to delineate their driving ideas.

### A medical academic's view

Marcia Angell is a senior lecturer in Social Medicine at Harvard Medical School and the former editor in chief of the NEJM. Recently, she wrote two articles in *The New York Review of Books* as reviews of Irwin Kirsch's new book *The Emperor's New Drugs: Exploding the Antidepressant Myth*, Robert Whitaker's *Anatomy of an Epidemic: Magic Bullets, Psychiatric Drugs, and the Astonishing Rise of Mental Illness in America*, and Daniel Carlat's *Unhinged: The Trouble With Psychiatry—A Doctor's Revelations About a Profession in Crisis*[13,14].

Dr Angell starts with a review of recent epidemiological data with misleading and false conclusions and continues to say that ‘nowadays treatment by medical doctors nearly always means psychoactive drugs…. In fact, most psychiatrists treat only with drugs.’ The truth is that there are no data to support Dr Angell's claim. The main argument is explicitly developed in the 5 March 2011 editorial of *The New York Times*, but still in the same editorial, it is clearly stated that psychiatrists earn more if they do psychotherapy while workload is less. It is well known among clinicians that throughout the world, private psychiatry is mainly based on psychotherapy, applied in a non-specific way. There is a very simple and obvious reason for this. Although later in her text, Dr Angell writes that ‘…If he sees three patients an hour for psychopharmacology, he calculates, he earns about $180 per hour from insurers. In contrast, he would be able to see only one patient an hour for talk therapy, for which insurers would pay him less than $100.’ Most psychiatrists working in the private practice around the world would do the calculation differently: if they are paid by the patient, then three patients four times per month make 12 sessions of psychotherapy vs. only three for psychopharmacology plus support. Unless one sees three patients per hour for 8 h every day for 20 days per month (480 individual patients), then he needs to utilize psychotherapy to fill the gaps in the appointments. The same number of 480 sessions is covered with only 40 individual patients under psychotherapy. Note that in many countries, this cost is out of pocket either completely or partially, which means the cost is freely negotiated between the therapist and the patient. It is clear that in contrast to what Dr Angell says, the psychiatric profession embraces because of the economic benefits psychotherapy and not psychopharmacology. On the other hand, in many countries, doctors are employed by the health care system and get fixed salaries for a fixed number of work hours. In this case, they choose psychotherapy because the workload is smaller as they can be responsible for one third of visits and a much reduced number of patients.

At another point, she writes that ‘Carlat does not believe that psychopharmacology is particularly complicated.’ But does a therapeutic method need to be ‘complicated’? It needs to be scientific. Scientific solutions are usually simple and often accused as being ‘simplistic’; this is a consequence and misunderstanding of the reductionistic method which constitutes the basis of the scientific approach. On the other hand, it is true that most clinicians (especially general practitioners) utilize psychopharmacotherapy in a simplistic and not well-informed way; training in biological therapies is also problematic worldwide and receives much less in time allocated in comparison to ‘talk’ therapies. In the authors' opinion, it is one of the oxymora of modern psychiatry, that is, to invest more on methods of unproven vs. proven efficacy.

Dr Angell repeats the argument that ‘instead of developing a drug to treat an abnormality, an abnormality was postulated to fit a drug.’ This argument has an inherent circular logic, and thus, it is almost impossible to argue against it logically, but it keeps emerging again and again in the writings of many authors. It is one thing to say that the boundaries of use of a specific medication are artificially expanded and another thing to say that one ‘invents’ a disease the drug supposedly cures. This is especially true when a drug has unpleasant side effects. It is as if one argues that it is not normal not to have nausea, tremor, headache, or extrapyramidal signs, and therefore, all people should receive those medications that cause them as side effects.

One important phrase by Dr Angell is the following: ‘Altogether, there were forty-two trials of the six drugs. Most of them were negative.’ This is false. Kirsch et al. received 47 trials from the FDA data, but only 35 (not 42) of them have data sufficient for analysis. Of course, most of the studies were not negative. The truth is that only in one of these 35 studies had been the effect size *d* in favor of placebo and was around zero in another five. In all of the remaining trials, it was above 0.10, and among them, 18 were above 0.30 [4,9,10]. The overall effect size was 0.32 which points to a non-perfect but clear superiority of the medication vs. placebo [4,9,10,15,16].

The most impressive of all statements follows: ‘even treatments that were not considered to be antidepressants—such as synthetic thyroid hormone, opiates, sedatives, stimulants, and some herbal remedies—were as effective as antidepressants in alleviating the symptoms of depression.’ At the end of her article, she concludes that ‘If we knew that the benefits of psychoactive drugs outweighed their harms, [and] that would be a strong argument, since there is no doubt that many people suffer grievously from mental illness. But as Kirsch, Whitaker, and Carlat argue convincingly, that expectation may be wrong.’ and that ‘Both psychotherapy and exercise have been shown to be as effective as drugs for depression, and their effects are longer-lasting.’ These arguments are evidently wrong [12,17,18] although, of course, different opinions exist [19]. Even according to Irwin Kirsch, all work through expectancy because they produce side effects, and thus, they ‘unblind’ the studies. In this way, the patient suspects that he is receiving an active drug instead of a placebo, his expectancy increases, and this boosts improvement. In simple words, this means that nothing works, and everything is placebo; this is exactly the opposite from ‘as effective as.’

However, Kirsch's suggestion is far from correct. As what recent meta-analysis has shown, the antidepressants which were more effective than others were those with a side-effect profile similar to placebo [20]. The ‘efficacy’ of non-antidepressant agents is a well-known artifact because of the problematic properties of the psychometric scales used and other methodological problems. For example, the Hamilton depression scale (HDRS) comprises a mixture of items; some of which reflect core symptoms of depression, but most reflect either non-specific symptoms like anxiety or sleep disorder while others might correspond both to depressive symptoms and to medication side effects (e.g., headache is both part of the anxiety-depressive symptomatology and also constitutes as a side effect of many drugs) [21]. In this frame, benzodiazepines could cause a significant improvement of HDRS total score, thus mistakenly suggesting they possess antidepressant effects. This has already happened with olanzapine who caused a significant reduction in the depressive score by improving sleep, agitation, and appetite, but not the core symptoms of depression in a study on bipolar depression [22]. This is probably one source of the response in the placebo arm since patients in that arm often receive benzodiazepines or other non-antidepressant agents with pronounced sedative or anxiolytic properties.

Later in her article, Dr Angell wonders whether ‘our drug-based paradigm of care, in some unforeseen way, be fueling this modern-day plague?’, and although the arguments are weak, she writes: ‘Whitaker's evidence is suggestive, if not conclusive (that medication changed the natural history of mental illness to the worse).’

It is also interesting that Dr Angell puts so much emphasis on the fact that ‘some (psychiatrists) embraced the new biological model, some still clung to the Freudian model, and a few saw mental illness as an essentially sane response to an insane world.’ This is not true. All over the world, the training of psychiatry is eclectic; stressing the fact that there is only one discipline which takes into consideration the developments of very different disciplines. On the other hand, even if one accepts the argument, it must be stressed that science was never an issue of voting or consensus, especially among people without research or academic background.

Concerning her comments on the development of the *Diagnostic and Statistical Manual of Mental Disorders, fifth edition* (DSM-V) of the American Psychiatric Association, she implies some kind of conspiracy theory: ‘These efforts to enhance the status of psychiatry were undertaken deliberately.’ Although criticism of the DSM has some value (e.g., concerning the validity and the number of diagnostic categories), again, she overstretches the interpretation of the situation: ‘DSM-III was almost certainly more “reliable” than the earlier versions, but reliability is not the same thing as validity.’ She fails to mention that reliability has the property to enhance validity by itself. High reliability implies the existence of some validity; low reliability implies lack of validity. And again, by overstretching the data, she suggests: ‘Not only did the DSM become the bible of psychiatry, but like the real Bible, it depended a lot on something akin to revelation. There are no citations of scientific studies to support its decisions.’ This is clearly wrong; however, it is beyond the scope of the current article to review the research support for the DSM classification.

### The philosophical-humanistic view

The second article was published in 1 March 2011 by Louis Menand, who is the Anne T. and Robert M. Bass professor of English at Harvard University; he has served as contributing editor at *The New York Review of Books* and is the author and editor of several books (his book *The Metaphysical Club* was awarded the 2002 Pulitzer Prize for History and the Francis Parkman Prize from the Society of American Historians). His article was published in *The New Yorker* under the title ‘Can psychiatry be a science?’ [23] as a comment on two new books, Gary Greenberg's *Manufacturing Depression* and Irwin Kirsch's *The Emperor's New Drugs*.

Menand writes that the psychiatric literature confuses the lay reader. He puts emphasis on the lack of consensus among psychiatrists, discusses the suspicion that the pharmaceutical industry is cooking the studies, and mentions that doctors prescribe antidepressants for patients who are not suffering from depression (eating disorders, panic attacks, premature ejaculation, and alcoholism). Clearly, it is more a lay person's view rather than a scientist in the field of medicine or mental health (which Louis Menand is not, of course). Throughout medicine, many medications are useful in the treatment of different disorders often unrelated (e.g., aspirin treats fever and pain and prevents atherosclerosis). This without taking into consideration that the mental disorders mentioned by Menand share clinical features and possibly etiopathogenesis. If Menand thought that these prescriptions are mistaken, he should have asked whether there are scientific data supporting it. Well, yes, there are such data available, but it is beyond the scope of the current article to discuss this in detail.

When discussing Greenberg's essentially anti-psychiatric approach, he utilizes an antiquated medical model which suggests a specific (single) cause and (direct) effect as well as a clear and qualitative boundary between health and disease. He makes this clear when he comments on the way both medicine and lay people deal with gastroesophageal reflux disease or ‘heartburn.’ He goes on to discuss depression in the frame of his medical model and wonders: ‘A fever is not a disease; it's a symptom of disease, and the disease, not the symptom, is what medicine seeks to cure. Is depression—insomnia, irritability, lack of energy, loss of libido, and so on—like a fever or like a disease? Do patients complain of these symptoms because they have contracted the neurological equivalent of an infection? Or do the accompanying mental states (thoughts that my existence is pointless, nobody loves me, etc.) have real meaning?’ Apart from the technical detail that fever is a sign and not a symptom, the truth is that pneumonia is a disease which can manifest alone or as the complication of other diseases, e.g., of lung cancer or AIDS. Infections might be also a side effect, e.g., of medication given to transplantation patients. The model he has in mind is too antiquated and too simplistic, but it reflects the lay person's or the non-expert's view of the issue. The problem is that this approach is misleading.

However, surprisingly, Menand notes by utilizing common sense: ‘Kirsch's conclusion is that antidepressants are just fancy placebos. Obviously, this is not what the individual tests showed. If they had, then none of the drugs tested would have received approval.’ Also, at another point, he says ‘for patients with very severe depression, the benefit of medications over placebo is substantial—which suggests that antidepressants do affect mood through brain chemistry. The mystery remains unsolved.’

He then discusses Kirsch's side-effect argument and says that ‘Kirsch has an answer: Cognitive Behavioral Therapy (CBT). He says it really works.’ As already mentioned, Kirsch's theory suggests that nothing works and everything is some kind of placebo. At another point, he says ‘Depressed patients in psychotherapy do no better or worse than depressed patients on medication.’ It is clear that he is not aware of the relevant literature [12,17,18].

Menand also mentions studies from 1949 suggesting low reliability of psychiatric diagnosis and some embarrassing political issues in the development of DSM (homosexuality, PTSD, self-defeating personality). Although these are real and important issues, they are discussed out of their frame with a strong tendency to over-generalize.

However, the most important part of his article is when he discusses what he believes is the real issue. Concerning existential problems and depression, he says ‘It's not even a problem that we should want science to solve for us.’ He discusses the bio-psycho-social model of mental disorders [24-27] by adding that ‘they have moral significance, since they involve us in matters such as personal agency and responsibility, social norms and values, and character, and these all vary as cultures vary.’ He continued that ‘The decision to handle mental conditions biologically is as moral a decision as any other…. Some people feel an instinctive aversion to treating psychological states with pills, but no one would think it inappropriate to advise a depressed or anxious person to try exercise or meditation.’

He then finishes his article with the phrase ‘…we don't want to be the kind of person who does not experience profound sorrow when someone we love dies? Questions like these are the reason we have literature and philosophy. No science will ever answer them.’

With these phrases, Menand summarizes the issue of the debate. It is philosophical-ideological. The word ‘want’ is central to this line of thought which in essence concerns ‘free will.’ According to it, medications suppress free will. It is not a matter of interpreting the data at all. Maybe one should encourage intellectuals interested in the free-will issue and mental illness to contact the homeless people in large cities. A significant number of them have a history of anxiety and depressive disorders that led them to the abuse of alcohol and other drugs and eventually led them to downfall.

### The ideological anti-psychiatric view

The third article was published on 27 February 2008, just after the Kirsch et al. meta-analysis was published, by Ann Blake Tracy, executive director of the International Coalition for Drug Awareness under the title ‘Has AntiDepressant Myth Bubble Burst?’ [28]. In this article, the British newspaper *The Guardian* is cited [29]. The article is a patchwork of issues and ideas directly or indirectly related to the antidepressant debate. It announces that ‘antidepressants have been all hype with no beneficial results for two decades. Along with that release, the British government announced that $335 Million would be allotted to train 3600 new talk therapists to help those suffering depression.’ Although this announcement was true, the authors of the current article did not manage to verify that it was related to a perceived antidepressant inadequacy by the British government.

Ann Blake Tracy then argues that antidepressants are similar to PCP or LSD by citing the study which suggested a therapeutic efficacy of ketamine in the treatment-resistant depression [30].

The rest of the article mentions parts of her 13 September 2004 testimony to the FDA where she said ‘Can you remember two decades ago when depressed people used to slip away quietly to kill themselves rather than killing everyone around them and then themselves as they do while taking SSRI antidepressants?’ At another point, she said ‘These are extremely dangerous drugs that should be banned as similar drugs have been banned in the past.’ In the same testimony, it is interesting how she argues about her scientific opinion on antidepressants: ‘All of these drugs produce dreaming during periods of wakefulness.’ Then, after connecting antidepressants with induction of manic symptoms (which although not proven beyond reasonable doubt, it is something most clinicians and researchers would accept), she describes what she thinks manic symptoms are: pyromania, kleptomania, dipsomania, nymphomania, and erotomania! Clearly this is closer to the eighteenth and early nineteenth century definition of mania as partial insanity than to modern understanding of mood disorders. Also, ‘Anyone who has witnessed someone in insulin shock would see the striking similarity to a violent reaction to an antidepressant.’ She also testified that ‘Child sex abuse has increased dramatically with even female teachers going manic on these drugs and seducing students.’

## Discussion

At the end of the day, the conclusion of all scientific data published so far suggests that antidepressants are effective and they treat depression; they do not make humanity happier. This is a key point that all the above-mentioned three articles miss. According to the most reserved, skeptical, and critical meta-analysis, antidepressants might not constitute the perfect silver bullet but are clearly superior to placebo, and their efficacy is superior to many established therapies of other specialties of medicine (e.g., cardiology and cardiosurgery) in terms of NNT [4,20,31]. They are also clearly superior to any psychotherapy when rigorous scientific methods are used for the comparison [12]. Apart from these meta-analyses, it is important to be cautious when interpreting the results of a meta-analytic study [32]. However, like any medication, their use needs to be cautious. Side effects, including the induction of the opposite pole in bipolar patients, even the induction of suicidality in specific populations, make necessary that antidepressants should be prescribed by trained physicians.

The status of psychiatry in medicine is highly complex. Even more complex is the way scientists outside mental health regard psychiatry. This seems to depend on each person's individual discipline and is probably influenced by ideological or religious aspects. Since psychiatry has limited exposure with publication of articles in general scientific journals, it is highly unlikely that the scientists' or even health professionals' opinion significantly differs from that of lay people, simply because they are not informed on the advances in the field of mental health.

A side effect of this limited exposure is that the debate on the modern face of psychiatry is essentially limited within psychiatry itself. In this debate, ‘hard science’ plays little role since the majority of psychiatrists and their training are oriented toward traditional psychosocial and ‘talk therapies.’ People outside psychiatry see that ‘psychiatrists disagree,’ and this is clearly reflected in the three articles reviewed here. The fact that it is essential as an argument even in the article by Marcia Angell reflects this limited exposure of psychiatry as a scientific discipline and part of medicine in highly reputable scientific and medical journals.

However, psychiatry is a field of medicine although, among medical specialties, psychiatry puts the greatest emphasis on psychosocial and personality determinants of the illness. When a patient comes to a psychiatrist with depression, he seeks alleviation of his suffering, and the physician is doing it to the best of his knowledge, mostly starting with adequate antidepressant treatment. This is also corresponding to the ‘free-will’ issue put forward by Menand, although in a different way. The patient can always choose not to seek help, although, based on a number of good information initiatives available, he should be aware that depression is a treatable disease.

The backbone of Marcia Angell's article is not supported by the data, not even Kirsch's work. The whole article is against psychiatry as a scientific medical field, in line with anti-psychiatric texts. It is similar to the article by Ann Blake Tracy, although it is more sophisticated in style. This latter article is based on the author's global impression, and it is poorly written. Menand is more balanced when dealing with the scientific reports, accepts that medication works at least in specific populations, but puts the issue in the frame of an ‘ethical’ and ‘existential’ dilemma, largely reflecting his view of human freedom and self-determination. He is somewhat embarrassed when facing the fact that although he does not really consider depression to be a health problem, still medication works, and he resolves it by suggesting a ‘moral’ maybe ‘omnipotent’ solution. It is a view more or less expected from an intellectual without expertise in medicine and psychiatry.

Overall, all articles show biased and incomplete approach of the issue. Still, they argue fiercely, and the authors show very much convinced on issues where experts struggle to interpret research data for years.

A number of arguments and lines of thought underlie all three articles. All stress that psychiatrists disagree, but in science, it has never been a matter of voting. They imply a ‘moral’ dilemma when using medication to alleviate mental symptoms. This is of course not new concerning the suffering of human beings. The dilemma of pain control in delivery was a hot issue some decades ago, as women should give birth in pain. In Genesis 3, it is written in words of the New International Translation: ‘To the woman He said, “I will greatly increase your pains in childbearing; with pain you will give birth to children. Your desire will be for your husband, and he will rule over you.”’ Nowadays, women are not ruled by anybody and make their choices at the delivery room: no analgesia, epidural or general anesthesia, or alternative methods such as hypnosis or relaxation. The same applies for the care of the terminally ill patients.

They set aside that morality has nothing to do with scientific data; they utilize an ideological and culturally biased interpretation of scientific data in a relativistic post-modern approach. It seems that prominent scientists, like when they join political parties and ideologies, frequently load their articles with their scientific prestige, but not with their scientific knowledge.

Why is this happening? According to Menand, ‘The critics who say that psychiatry is not really science are not anti-science themselves. On the contrary: they hold an exaggerated view of what science, certainly medical science, and especially the science of mental health, can be.’ The authors of the current article strongly disagree. The kind of critique psychiatry faces is inappropriately negativistic and selective in which aspect of the data to pick. It seems ideologically and philosophically fueled and exploits what in the philosophy of science is obvious: it is easier to argue that something is not true than it is. This is a side effect of falsificationism; however, falsificationism uses critique to the benefit of progress, not as a self-sustained and self-propelled negativistic and rigid stance and way of viewing things. Only genetics and Darwinian theory are under similar ideological fire, maybe because, like psychiatry, both put a big question mark on the existential question of human free will and existence.

Indeed, the issue of free will is a hot topic in philosophy which strives to follow the advances in neuroscience. It is beyond the scope of the current article to discuss free will; however, it should be noted that a stream of thought suggests that ‘unless our choices are ultimately uncaused they cannot be free’ [33]. This concept makes no sense in science; when events have ‘no cause,’ they are random, and free will does not mean one throws dices all the time. Most ‘philosophers’ cannot understand or do not know at all that the human brain is not a Turing machine; it is a more complex computational system which does not follow the rules of informatics science the way we conceive them today (of course, there are some theoretical proposals like the ‘quantum computers,’ etc.). However, although unsound, these philosophical approaches unfortunately have an important consequence since they are radically and inherently hostile to the concept of mental health and disease and thus to psychiatry. To some extent, they might tolerate ‘talk therapies,’ but for psychopharmacology, this is impossible.

It is to be noted that none of the three articles or any newspaper or media say anything concerning the huge economical cost of alternative therapies (including most psychotherapies), which are culturally and philosophically accepted and are based on tradition; however, only a small minority of these techniques has some, but not complete, support by hard evidence (in sharp contrast to medication), and even then, their efficacy is lower than that of medication treatment [17,18] and might not be better than placebo at all [12]. Also, no mentioning is made of the fact that general practitioners and not psychiatrists prescribe most antidepressants and treat most psychiatric patients. Whether they are adequately trained to do so or not, it is an ongoing debate; however, the blame for this should not be put on the discipline of psychiatry.

While authors argue whether depression is a medical problem and whether and how antidepressants work, they miss the fact that a number of conditions (e.g., myopia) do not fulfill their criteria for a medical disorder. It is interesting that similar criticism has been made to the use of correction glasses, and there are authors suggesting that they worsen short-sight problems in the long term [34]. This ideological and romantic view of health is not only embarrassing when facing cosmetic surgery, the use of viagra, or even preventive medicine aiming at prolonging life (is there anything more natural than getting older and die?), but this line of thought is also similar to suggesting that the use of X-ray examination increased the incidence of bone fractures or that the use of higher Tesla MRI equipment increased the incidence of vascular encephalopathy and cancer metastasis. In this frame, the demand posed (directly or indirectly) by Dr. Angell that for one to be considered as a medical condition (‘disease’) that it should include abnormal laboratory testing is misleading. After all, response to medication should be considered to be a strong biological marker for depression (however, the whole debate concerns the efficacy of these agents).

There is no better conclusion of the present article than what is impressively written by Menand: ‘Science, particularly medical science, is not a skyscraper made of Lucite. It is a field strewn with black boxes. There have been many medical treatments that worked even though, for a long time, we didn’t know why they worked—aspirin, for example. And drugs have often been used to carve out diseases. Malaria was “discovered” when it was learned that it responded to quinine. Someone was listening to quinine.’

As long as psychiatry is a neglected field in the medical literature, the gap will be filled by the lay media or the ‘lay-like’ articles of non-lay authors, with adverse consequences on public health.

### Louis Menand^a^ responds

Even though there are four of them, the authors manage to completely misinterpret what I wrote. My article was not anti-science, anti-psychiatry, or anti-psychopharmacology. On the contrary: it was a defense of psychiatry, including psychopharmacology, against most of its critics. The authors seem to take offense even at summaries of criticisms of psychiatry, which is what most of my article consists of. I was not endorsing the views that I tried to explain. I certainly don't endorse Kirsch's book, which seems to be the main target of the authors' animus. And I don't know where the authors got the idea that I think taking medication suppresses free will, since I specifically refuted that supposition. Free will and determinism may be, as the authors say, a ‘hot topic,’ but I have no interest in it.’

Surely the authors would agree that psychiatry is a field that has undergone unusually drastic and often highly publicized paradigm revisions in the last fifty years, accompanied by an enormous amount of criticism from within the field. A non-scientist might well feel confused, and might even wonder whether the present state of knowledge is due for an overhaul, as well. I was addressing that person. The authors often use the term ‘ideological’ to discredit arguments that they believe to be unscientific or anti-science. This is specious. Medicine, like everything else we do, is practiced within an intellectual context. Trying to understand such contexts is what English professors do.

## Endnote

^a^Louis Menand: Correspondence: Department of English, Harvard University, 12 Quincy St, Cambridge, MA 02138, USA; Email: menand@fas.harvard.edu.

## Competing interests

KNF is a member of the International Consultation Board of Wyeth for desvenlafaxine, BMS for aripiprazole in bipolar disorder, and Servier for agomelatine; he has received honoraria for lectures from AstraZeneca, Janssen-Cilag, and Eli Lilly and research grants from AstraZeneca and Pfizer Foundation. CH was a study coordinator for Servier and has received grants from Eli Lilly. He is in the advisory board for BMS and Janssen-Cilag; gave lectures to Lupin, Eli Lilly, Janssen-Cilag, and Medicom; and is a faculty member at LINF. HJM has received grants and is a consultant for and on the speakership bureaus of AstraZeneca, Bristol-Myers Squibb, Eisai, Eli Lilly, GlaxoSmithKline, Janssen-Cilag, Lundbeck, Merck, Novartis, Organon, Pfizer, Sanofi-Aventis, Schering-Plough, Schwabe, Sepracor, Servier, and Wyeth. SK has received grants/research support from Bristol-Myers Squibb, Eli Lilly, GlaxoSmithKline, Lundbeck, Novartis, Organon, Pfizer, Sepracor, and Servier. He is currently serving or has served as a consultant/advisory board for AstraZeneca, Austrian National Bank (OENB), Bristol-Myers Squibb, Eli Lilly, German Research Foundation (DFG), GlaxoSmithKline, Janssen, Lundbeck, Merck Sharp and Dohme (MSD), Organon, Pfizer, Schwabe, Sepracor, and Servier and a member of the supervisory boards of universities. He is currently or formerly with the speaker's bureau for Angelini, AstraZeneca, Bristol Myers Squibb, Eli Lilly, Janssen, Lundbeck, Merck Sharp and Dohme (MSD), Organon, Pierre Fabre, Pfizer, Schwabe, Sepracor, and Servier. JLI has received speaker's honoraria, travel grants or consultancy fees from Eli Lilly, Bristol-Myers Squibb, Lundbeck, Pfizer, and Servier and is currently or formerly a member of the advisory boards of Eli Lilly, Pfizer, and Bristol-Myers Squibb, and he is a faculty member of the Board of the Lundbeck Institute (Lundbeck Neuroscience Foundation) of Copenhaguen.
